# Effects of 12-Week Infant Shantala Massage Program on Maternal Emotional Well-Being Following First-Time Birth

**DOI:** 10.3390/healthcare13151895

**Published:** 2025-08-03

**Authors:** Anna Gogola, Rafał Gnat

**Affiliations:** Institute of Physiotherapy and Health Sciences, Academy of Physical Education, 40-065 Katowice, Poland; aniagogla@op.pl

**Keywords:** postpartum, depression, massage, complementary therapy

## Abstract

**Background/Objectives:** This study aimed to determine whether postpartum mothers exhibit a uniform trajectory of postpartum emotional status (PES) changes or if distinct subgroups with differing trajectories of PES exist. Additionally, it investigated whether intensified tactile stimulation of the infant through Shantala massage influences maternal PES. **Method:** A quasi-experimental design with a matched control group was employed. Eighty women following their first physiological delivery volunteered to participate. The intervention involved applying intensified tactile stimulation to the infant via Shantala massage over a 12-week postpartum period. Maternal PES, divided into negative and positive emotional domains, was assessed using four standardized questionnaires. **Results:** Two opposing trajectories of PES change were identified: adverse and favorable. Intensified tactile stimulation was associated with improvement in maternal emotional status along both trajectories. **Conclusions:** PES changes do not follow a uniform course across all women; notably, those with a favorable trajectory often begin with more severe symptoms. Overlooking this distinction in diagnosis, prevention, and treatment may result in suboptimal care. The factors influencing PES trajectories remain unidentified but may affect clinical intervention outcomes. The Shantala massage intervention appears to slow the progression of emotional disorders in women with adverse PES changes and accelerate recovery in those with favorable changes. Implementation of this approach in clinical settings is recommended.

## 1. Introduction

Pregnancy and the postpartum period involve substantial transformations across multiple domains of a woman’s functioning, including marked emotional adaptations. The scientific literature has predominantly focused on the negative aspects of postpartum emotional status (PES), particularly postpartum depression (PPD) [1]. Global data on PPD are concerning, with prevalence estimates ranging from 6.5% to 25.8% [2], and current studies suggesting that up to 34% of mothers may experience depressive symptoms [3]. According to the World Health Organization, depression is projected to become the most common disease globally by 2030 [4]. It is important to emphasize that the most serious consequences of PPD extend well beyond the immediate postpartum period, potentially affecting the child into adulthood—for example, through the development of insecure attachment patterns [5,6]. In severe cases, PPD may endanger both maternal and child well-being, contributing to thoughts of harming the child [4], suicidal or self-injurious behaviors [5,7], and postpartum psychosis [8].

While recognizing negative emotional states during the postpartum period remains crucial, recent research has increasingly emphasized the importance of positive emotional experiences as well. Low positive affect has been identified as a key distinguishing feature of depression, highlighting the need to assess both the negative and positive domains of PES [9]. Evidence suggests that individuals who experience positive emotions more frequently tend to exhibit greater “positive emotionality,” which may serve as a protective factor against emotional distress [10]. Similarly, two recent meta-analyses of positive psychological interventions—conducted in different populations—demonstrated moderate improvements in positive affect and reductions in negative affect among individuals with depression [11], as well as enhanced well-being and decreased symptoms of ill-being among young people [12]. This dual-domain framework, which integrates both positive and negative emotional dimensions, is increasingly acknowledged as essential for a comprehensive and nuanced understanding of PES and its evolution over time [13,14].

Despite growing interest in this area, substantial gaps remain. In particular, it is still unclear whether early PES patterns can reliably predict future emotional trajectories, or whether distinct subgroups of mothers follow divergent pathways—some showing improvement, while others experience deterioration. Identifying these emotional vectors could fundamentally reshape early screening strategies, inform the timing and nature of interventions, and enhance long-term approaches to maternal mental health care.

Identifying effective preventive and therapeutic strategies is particularly crucial for women exhibiting adverse trajectories of PES. To date, several interventions have demonstrated beneficial effects, including aerobic exercise [15,16], behavioral sleep interventions for infants and mothers [17], psychosocial peer support, nondirective counseling, group-based therapies [2], and pharmacological treatment—primarily selective serotonin reuptake inhibitors as a first-line option [18]. However, there remains a pressing need for accessible, acceptable, and low-risk interventions. In this context, complementary manual therapies such as intensified tactile stimulation—specifically Shantala massage—warrant greater attention. While pharmacologic and psychosocial treatments have proven efficacy, they may not be feasible or desirable for all women. In contrast, mother-administered infant massage represents a simple, low-cost, non-invasive, and intuitive alternative with substantial potential. Emerging evidence suggests that intensified tactile stimulation not only benefits the infant, but also has a measurable positive impact on maternal emotional well-being [19,20,21,22,23].

In light of the identified gaps in knowledge surrounding PES, the following research questions were formulated: (1) Do mothers following their first physiological delivery exhibit a uniform trajectory of PES changes, or do distinct subgroups with differing trajectories exist?; (2) How does PES change within these distinct subgroups over time?; (3) What is the effect of intensified manual tactile stimulation of the infant—specifically through Shantala massage—on PES trajectories within these subgroups?

## 2. Materials and Methods

### 2.1. Design

This quasi-experimental study employed a non-randomized design due to ethical considerations. The intervention—Shantala massage—was implemented in an experimental group of volunteer participants. The control group was derived from participants in a previous study that followed an identical methodology but without the intervention, using a matched sampling procedure (allocation ratio 1:1). Measurements of the dependent variables were collected at two time points: on the third day postpartum and during the 13th postpartum week. The study protocol was approved by the institutional Research Ethics Committee (Approval No. 8/2017).

### 2.2. Participants

The minimum required sample size was determined based on pilot study data using a standard sample size calculator [24], with the following assumptions: α = 0.05, a minimum detectable effect size of 5%, and an anticipated dropout rate of 20% due to the high likelihood of exclusion criteria being met. The largest sample size requirement was associated with the Positive and Negative Affect Schedule scale, which indicated a minimum of 42 participants. To accommodate planned subgroup analyses and account for potential attrition, recruitment continued until this number was doubled and the expected dropout margin added, yielding a final recruitment target of 101 participants.

Participants were recruited during routine postpartum follow-up visits in the Obstetrics Department between January 2021 and April 2022. Eligibility was confirmed by a multidisciplinary team consisting of a medical doctor, physiotherapist, and psychologist, based on clinical interviews and assessments.

The inclusion criteria were as follows: age between 18 and 40 years; first physiological childbirth; no history of miscarriage; natural conception; full-term delivery; uncomplicated pregnancy; normal fetal development confirmed by ultrasound; absence of known risk factors for depression (including maternal disability, substance or alcohol abuse, suicidal or self-harming tendencies, low socioeconomic status, relationship violence, or lack of support during pregnancy [4]), and absence of other former or current psychological/psychiatric diagnoses (either or not requiring medication). The group of 119 women was selected. All signed their informed consent. During the perinatal period (within 3–5 days postpartum), participants were screened for the following exclusion criteria: Apgar score < 8; complications during delivery associated with compromised maternal health (e.g., thrombosis, pulmonary embolism, postpartum hemorrhage, or infection); and deterioration in the infant’s condition requiring hospitalization beyond the specified perinatal period. All assessments were conducted by a medical doctor. Throughout the 12-week observation period, participants were continuously monitored for additional exclusion criteria, including any condition resulting in separation between mother and newborn for more than one week (e.g., hospitalization of the mother or child, or legal custody issues); deterioration of the mother’s health requiring hospitalization for more than one week and/or preventing continuation of the experimental intervention for more than one week; and significant deterioration in the mother’s PES due to acute, non-study-related events, such as financial crisis (e.g., job loss of the primary income provider and/or ≥50% reduction in household income), traumatic family events (e.g., death or serious illness of a family member, abandonment by the child’s father, or legal proceedings affecting parental rights). These latter factors were identified through regular psychological evaluations. Participants were also excluded if they failed to adhere to the intervention protocol, defined as completing fewer than 50% of the planned intervention sessions. Some of the exclusion criteria involve subjective or potentially post hoc judgments, which may introduce bias. To minimize this risk, decisive assessments were carried out jointly by a medical doctor, psychologist, and physiotherapist. Decisions regarding exclusion were made by consensus during team meetings to ensure consistency and reduce individual bias.

**Figure 1:**
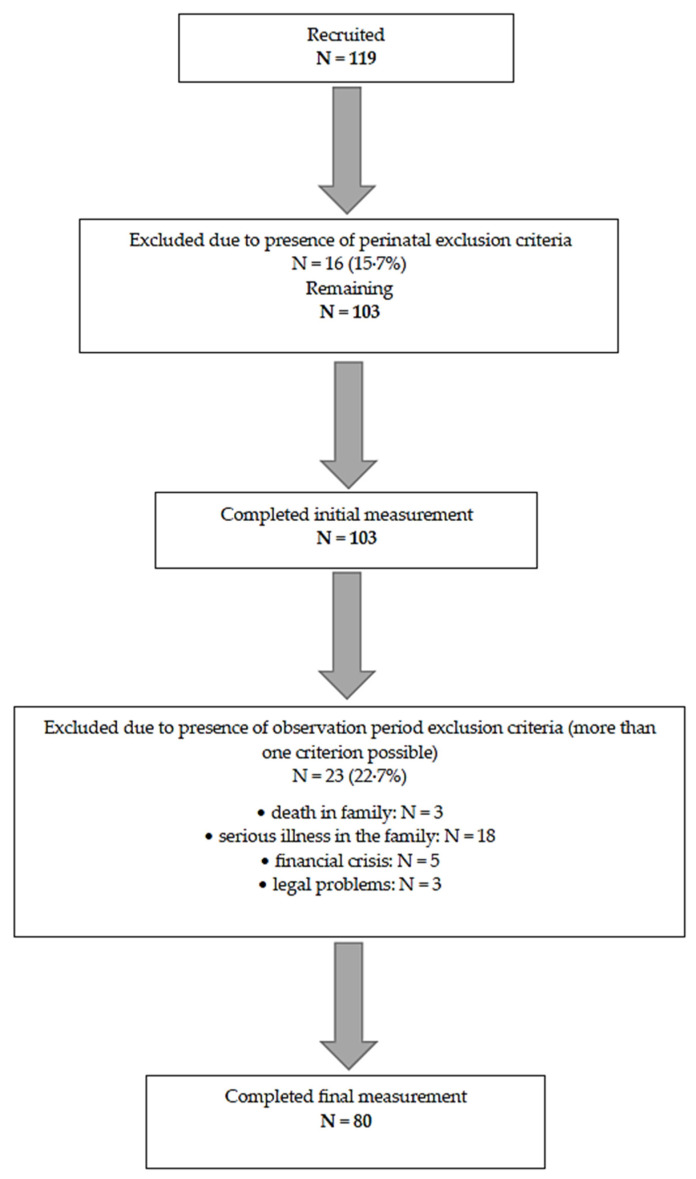
Participants flow through the consecutive stages of the procedure.

Immediately following delivery, 16 women were excluded from the study. The initial measurement of dependent variables was completed by 103 participants. During the 12-week observation period, an additional 23 participants were excluded, resulting in a final sample of 80 complete data sets (see Figure 1, Table 1).

For each woman who completed the final assessment, a matched counterpart was selected from participants in a previous study conducted using the same methodology [25]. This matching procedure could only be performed after the cluster analysis of the data, which is described in detail in the subsequent sections.

### 2.3. Measurements

To investigate PES across both emotional domains—negative and positive—four standardized questionnaires were employed. The negative emotion domain was assessed using the Edinburgh Postnatal Depression Scale (EPDS), the Hospital Anxiety and Depression Scale (HADS; comprising depression and anxiety subscales), and the negative affect subscale of the Positive and Negative Affect Schedule (PANAS). The positive emotion domain was evaluated using the positive affect subscale of the PANAS and the Parenting Sense of Competence Scale (PSOC), which includes satisfaction and effectiveness subscales (see Table 2). For all scales and subscales, the interpretation of scores is consistent: higher scores reflect a higher intensity of the measured emotional state. Thus, in the negative emotion domain, higher scores indicate a worsening emotional condition, whereas in the positive emotion domain, higher scores indicate an improvement in the participant’s emotional state.

### 2.4. Procedure

No earlier than one month prior to delivery, participants underwent a five-hour training session focused on the experimental intervention. This included both theoretical instruction and practical training in the Shantala massage technique, using infant mannequins. Participants were also provided with supplementary multimedia materials to reinforce learning.

On the third day postpartum, the initial measurement of the dependent variables was conducted. Participants completed a series of standardized questionnaires by reading each item on a computer screen and entering their responses. In total, 61 items were presented. To maintain concentration and reduce fatigue, five-minute breaks were scheduled between each questionnaire. The entire procedure lasted approximately one hour.

**Figure 2:**
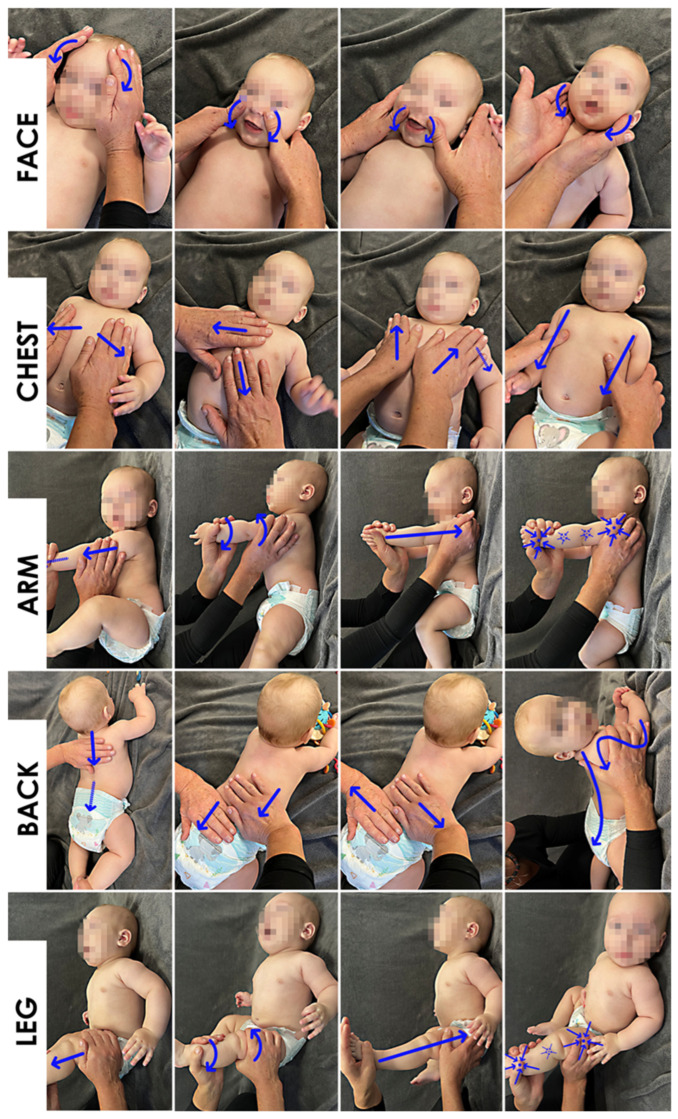
Intensified tactile stimulation of the infant using the Shantala massage technique. Five body regions were distinguished for the procedure: face, chest and abdomen, upper limbs, back, and lower limbs (organized in rows). For each region, four specific massage techniques were applied (organized in columns). Beginning on the third day postpartum, participants administered the massage in the prescribed sequence twice daily—once in the morning and once in the afternoon—for approximately 14 min total (2 min per region). Additional spontaneous sessions, based on maternal discretion, were permitted. The intervention concluded at the end of the 12th postpartum week. Written informed consent for publication was obtained from the parents.

The next stage encompassed the 12-week postpartum period. In the experimental group, an intervention was implemented in the form of intensified manual tactile stimulation of the child, using the Shantala massage technique administered at home [34]. For this purpose, five body regions were defined: face, chest and abdomen, upper limbs, back, and lower limbs. Four manual techniques were applied to each region. Beginning on the third day after delivery, participants performed the massage in the specified sequence twice daily—once in the morning and once in the afternoon—for approximately 14 min per session (2 min per region). Additional spontaneous sessions, initiated at the mothers’ discretion, were permitted. The intervention concluded at the end of the 12-week postpartum period (see Figure 2; informed written consent was obtained from all parents).

Throughout the intervention period, participants completed a daily online diary to record the number of massage sessions performed and to monitor for the occurrence of any postpartum exclusion factors. Monitoring for exclusion criteria was further supported by routine monthly consultations with a medical doctor, physiotherapist, and psychotherapist. Additional on-demand consultations were available upon participants’ request. To enhance adherence to the intervention protocol, participants received a standardized daily text message reminder: “Don’t forget the massage and the diary.”

Within the 13th week postpartum, the final measurement of the dependent variables was performed during the follow-up visit in the hospital. The procedure was exactly the same as during the initial measurement.

The 12-week postnatal period was chosen for observation, as this is the time of the most intense emotional changes in women. After 12 weeks, the emotional condition is relatively stabilized [35]. Repeating the same testing procedure after 12 weeks may introduce recall bias. To minimize this risk, we used standardized, validated questionnaires with structured items that assess current emotional states rather than requiring retrospective comparisons. Participants were not informed in advance that the same instruments would be re-administered, reducing the likelihood of recall-driven responses. Furthermore, the 12-week interval between assessments was sufficiently long to mitigate short-term memory effects and to obtain a measurable change in PES [36].

### 2.5. Data Management

All responses were recorded in a secure computerized database. To prevent potential bias, the authors did not access the database until data collection was fully completed. Final scores for all scales were computed according to the original scoring instructions and subsequently used for statistical analysis.

### 2.6. Statistical Analysis

To assess the internal consistency of the questionnaire data, Cronbach’s alpha coefficients were calculated. The Shapiro–Wilk test was employed to evaluate deviations from a normal distribution. To identify subgroups within the experimental group exhibiting distinct trajectories of PES changes, hierarchical cluster analysis was conducted using Ward’s case agglomeration method and Euclidean inter-cluster distance. This unsupervised approach was selected because it allows for the data-driven identification of latent subgroups based on PES trajectories without requiring predefined classification criteria. Intra-group comparisons were performed using the paired Student’s t-test or the Wilcoxon signed-rank test, depending on data distribution. Inter-group comparisons were conducted using the independent t-test or the Mann–Whitney U test, as appropriate. All statistical analyses were carried out using Statistica 13.0 (StatSoft, Tulsa, OK, USA), and all analyses adhered to the original group assignments.

## 3. Results

### 3.1. Internal Consistency

All scales and subscales demonstrated acceptable internal consistency, with Cronbach’s alpha coefficients ranging from 0.70 to 0.90.

### 3.2. Cluster Analysis

**Figure 3:**
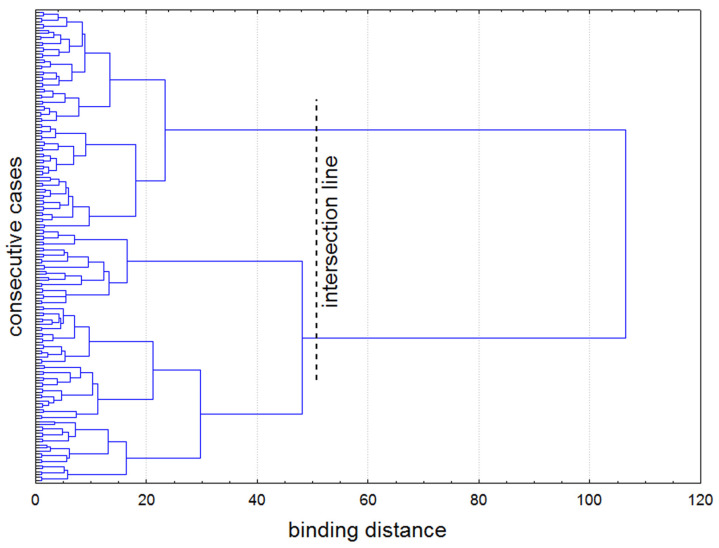
Simplified dendrogram graph showing case agglomeration process in the cluster analysis. The input data were the standardized final scores of all scales and subscales. Agglomeration was conducted according to the Ward method with the Euclidean inter-cluster distance. Both visual analysis and the Mojena method clearly indicate the optimal location of the intersection line cutting the longest branches of the dendrogram at the binding distance (dimensionless) ~50.

The dendrogram graph revealed two distinct subgroups of participants. The point of separation (cut-off line) was determined through visual inspection and confirmed using the Mojena method (Figure 3). No significant differences were found between the subgroups with respect to demographic characteristics (Table 3). We refer to these subgroups as the adverse PES trajectory subgroup and the favorable PES trajectory subgroup, based on the theoretical framework introduced earlier [25]. Briefly, the adverse trajectory is characterized by an overall decline in emotional status reflected by lower scores in the questionnaires related to the domain of positive emotions and higher scores in the questionnaires related to in the domain of negative emotions. In contrast, the favorable trajectory reflects an improvement in emotional status, with increasing positive emotion scores and decreasing negative emotion scores.

### 3.3. Control Group Selection

To establish a comparable control group, a matched sampling procedure was employed. In a previous project conducted using identical methodology aside from the intervention, data were collected from 134 participants. For each participant in the experimental group, a matched counterpart was selected from previous study subjects using a non-returnable computer algorithm, based on the following hierarchical criteria: (1) identical PES trajectory (adverse or favorable), (2) minimal age difference, and (3) minimal difference in body mass index. The resulting experimental and control groups were statistically equivalent (see Table 4).

### 3.4. Descriptive Statistics and Analysis of Differences

Initially, the analysis of differences was conducted without dividing participants into subgroups based on adverse or favorable PES trajectories. Under these conditions, a relatively clear pattern emerged. The experimental and control groups exhibited comparable baseline scores, with the exception of the PSOC effectiveness subscale (Table 5). Over the course of the observation period, the experimental group demonstrated significant intra-group improvements across all measures. By the end of the 12-week period, this group showed significantly better outcomes than the control group, with all inter-group differences reaching statistical significance in favor of the experimental group.

Following the division into subgroups based on adverse and favorable PES trajectories, a markedly different picture emerged. It should be noted that these subgroup analyses carry reduced statistical power and should be interpreted as exploratory. Among participants exhibiting adverse PES changes, emotional status deteriorated in both the experimental and control groups (Table 6). These groups began at comparable baseline levels, with significant inter-group differences observed only for the EPDS. In the experimental group, despite the implementation of the intervention, participants’ emotional status either remained stable or slightly worsened. Some improvement was observed in the domain of positive emotions (specifically in the PANAS positive affect and PSOC effectiveness subscales). In contrast, participants in the control group experienced significant deterioration across all measures, with all intra-group differences reaching statistical significance. At the final assessment, the control group showed significantly worse outcomes than the experimental group, with all inter-group differences favoring the latter.

In contrast, among participants demonstrating a favorable PES trajectory, emotional well-being improved in both the experimental and control groups (Table 7). At baseline, the experimental group exhibited slightly poorer scores, with significant inter-group differences noted for HADS and PSOC effectiveness. Nevertheless, both groups showed significant improvement over time. Scores in the domain of negative emotions decreased, while scores in the domain of positive emotions increased, with all intra-group differences reaching statistical significance. However, the magnitude of improvement was greater in the experimental group, which demonstrated significantly larger gains across all scales except the EPDS and the positive affect subscale of the PANAS. At the final assessment, all statistically significant inter-group differences favored the experimental group.

**Figure 4:**
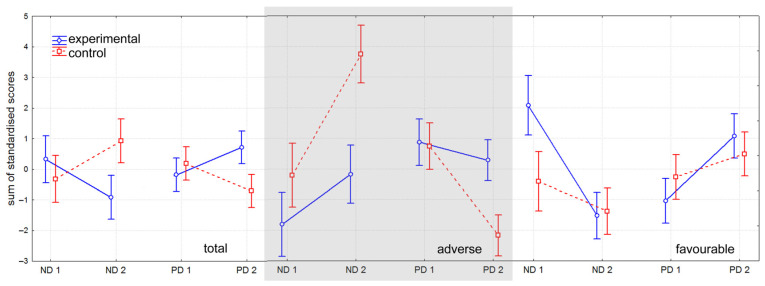
Summarized standardized scores (±standard deviations) of all questionnaires. ND—domain of negative emotions; PD—domain of positive emotions; 1—initial measurement; 2—final measurement. Presented are results for the total group of participants (left) as well as for the identified subgroups of adverse (middle) and favorable (right) trajectory of postnatal emotional transformation. In the total group, an impression occurs that there is a deterioration in the condition of all participants, expressed by an increase in the summarized score in ND and a decrease in PD. After subgroups are distinguished, it becomes clear that only the adverse trajectory of postnatal emotional transformation is linked to deterioration of participants’ condition. Favorable trajectory results in an improvement of the emotional status.

To provide a clear summary of the findings, standardized scores from all questionnaires were aggregated. This approach reinforced the initial impression that, when subgroups are not considered, the emotional condition of participants in the control group appears to deteriorate, while that of the experimental group improves (Figure 4, left). However, when changes are examined separately by PES trajectory subgroups, a more nuanced picture emerges. Among participants in the adverse PES trajectory subgroup, emotional well-being declined in both groups, but the decline was less pronounced in the experimental group (Figure 4, middle). Conversely, in the favorable PES trajectory subgroup, all participants demonstrated improvement, though the extent of improvement was greater in the experimental group than in the control group (Figure 4, right). These summary results are presented solely to illustrate overarching trends; no statistical tests were conducted on these aggregated values.

## 4. Discussion

The results of the present study generally align with previous findings indicating that intensified manual tactile stimulation of an infant, administered by the mother, influences not only the child but also the mother’s emotional well-being. For instance, Márquez-Doren et al. [19] demonstrated that infant massage facilitates the psychosocial transition to motherhood and reduces maternal fears and uncertainties regarding parenting skills. Dehkordi et al. [20] found that mothers who performed 15 min infant massages twice daily for six weeks experienced a significant reduction in PPD incidence. Higgins et al. [21] reported beneficial effects of infant massage classes for mothers with PPD. Similarly, Onozawa et al. [22] randomized 34 women to receive either infant massage combined with support classes or support classes alone, observing decreased depression levels in both groups. Moussa et al. [23] provided evidence that infant massage affects oxytocin release bidirectionally in both mothers and infants, linking maternal postpartum behaviors to hormonal regulation. Intensified tactile stimulation of the infant thus represents a promising preventive approach for postpartum maternal emotional disorders. Its advantages over pharmacological or psychological interventions include low cost, minimal time requirements, ease of use following brief training, and alignment with mothers’ natural caregiving behaviors such as stroking and hugging. Nevertheless, further research is required to comprehensively document its impact on postpartum emotional status.

The key and novel finding of this study is the identification of two distinct and opposing trajectories of PES changes in women following their first physiological delivery. Interestingly, and somewhat counterintuitively, a poorer emotional state immediately postpartum does not necessarily predict a negative outcome. It is the subgroup exhibiting the adverse PES trajectory—characterized by a relatively mild initial presentation—whose condition progressively deteriorates over time. Conversely, the favorable trajectory, despite being associated with more severe initial PES symptoms, shows improvement as time progresses. Crucially, these divergent patterns are not apparent when analyzing either the experimental or control group separately, indicating that analyses conducted without subgroup stratification may lead to misleading conclusions. Furthermore, these trajectories were identified even among women with a relatively low risk for depression, as ensured by stringent selection criteria.

Discovering the determinants of these two scenarios of PES would be of invaluable importance. Based on our results, we can only conclude that these are not factors: age, body morphology, level of education, or pregnancy planning (Table 3). Most probably the background will be multifactorial [5,37,38] In further research, the authors recommend paying attention to the relationships between the trajectories of PES changes and chemical/hormonal factors [38,39,40].

Our findings have the potential to fundamentally reshape how clinicians approach the prevention and management of postpartum emotional disorders. Additionally, many previous studies might warrant reevaluation if different trajectories of PES changes had been taken into account (e.g., [41]). Importantly, these trajectories may be predicted early using simple, cost-effective, and time-efficient questionnaires. A critical, yet unresolved, question remains regarding the optimal cut-off points for these tools to ensure reliable prognostic use. Addressing this issue will be the focus of our future research.

The intervention employed in this study—intensified tactile stimulation using Shantala massage—should be considered unequivocally beneficial. The existing literature has documented the positive effects of various forms of tactile stimulation on maternal well-being, including kinesthetic stimulation, infant massage, and baby massage [42], with some evidence extending to other family members as well [43]. The impact of Shantala massage appears similarly advantageous. In cases characterized by adverse PES trajectories, the massage was found to slow the progression of emotional disturbances, though without producing measurable improvement. Conversely, in participants demonstrating favorable PES trajectories, emotional improvement occurred even in the control group, suggesting the involvement of additional, unidentified factors. Future research should aim to identify these contributing factors. Importantly, the application of Shantala massage significantly accelerated the rate of this emotional improvement. We therefore advocate for its broader use in both clinical and home settings due to its clear benefits: it is simple to perform, requires only a few hours of training, is cost-effective, minimally time-consuming, and does not require any specialized equipment. Additional benefits for the infant have also been well-documented for similar forms of intervention [42,44]. However, it is essential to precede the home-based application of the massage by proper training and to provide professional supervision ensuring safe and effective implementation.

Our study presents several limitations. First, it employed a non-randomized design. A quasi-experimental approach with matched sampling was chosen to prevent the ethical issue of denying a potentially beneficial intervention to a subset of participants solely due to random assignment. Under these circumstances, matched sampling for the control group was deemed the optimal method; however, it must be kept in mind that even rigorous matching cannot entirely eliminate the possibility of unmeasured confounding variables, which may have influenced the observed outcomes. Second, the generalizability of our findings is limited by the study population, which consisted solely of primiparous women following physiological childbirth and meeting relatively strict selection criteria. As such, our results may not be applicable to more diverse maternal populations, including multiparous women or those with pre-existing emotional or psychiatric conditions. Future research in these groups is necessary to evaluate the robustness and broader applicability of our findings. Third, our reliance on self-reported outcomes introduces the possibility of reporting bias, including underreporting or overreporting of emotional states due to social desirability, recall inaccuracies, or subjective interpretation of questionnaire items. Although we used standardized, validated instruments, self-report measures remain inherently susceptible to bias. Incorporating objective clinical assessments or multi-informant reports in future studies may enhance measurement validity and strengthen conclusions. Fourth, the study experienced a relatively high dropout rate, which can be attributed to the heightened vulnerability of participants during the postpartum observation period. This limitation was addressed in advance by incorporating an estimated 20% dropout rate in the sample size calculation. Taken together, while the current study provides promising evidence regarding the effects of Shantala massage on maternal emotional well-being, the findings should be interpreted with appropriate caution.

## 5. Conclusions

Changes in PES do not follow a uniform trajectory among all women; instead, two opposing vectors—adverse and favorable—are observed. Interestingly, and contrary to intuition, the favorable trajectory often begins with a more severe initial emotional state. Failure to account for this distinction may result in misguided preventive or clinical interventions.

The direction of the PES vector appears to be independent of age, body morphology, educational level, or whether the pregnancy was planned. This suggests that other, yet unidentified, factors may influence emotional outcomes and modulate the effectiveness of interventions.

Shantala massage demonstrated several beneficial preventive effects. In women exhibiting an adverse PES trajectory, it appeared to slow the progression of emotional distress. In those with a favorable trajectory, it seemed to accelerate emotional improvement.

## Figures and Tables

**Table 1:** Demographic characteristics of the experimental group.

	Mean ± SD (Min–Max) or Number (%)
age (years)	30.95 ± 4.58 (20.00–40.00)
body height (m)	1.67 ± 0.06 (1.55–1.80)
body mass index (kg/m^2^)	27.51 ± 2.48 (22.86–33.46)
	secondary: 14 (17.50)
education level	high: 66 (82.50)
	yes: 14 (17.50)
pregnancy planning	no: 66 (82.50)

**Table 2:** Summary of all utilized questionnaires and their subscales.

	Subscale	Maximal Score (Points)	Interpretation	Polish Adaptation Validity Metrics
The Edinburgh Postnatal Depression Scale [26]	none	30	≥13 = risk of depression	Cronbach’s alpha 0.91 sensitivity 96% specificity 93% [27]
The Hospital Anxiety	depression	21	≥11 = severe disorder	Cronbach’s alpha 0.85 [28]
and Depression Scale [29]	anxiety	21	≥11 = severe disorder	Cronbach’s alpha 0.81 [28]
Positive and	negative affect	50	≥20 = strong affect	
Negative Affect Schedule [30]	positive affect	50	≥30 = strong affect	Cronbach’s alpha 0.86 [31]
Parenting Sense	satisfaction	54	≥35 = high satisfaction	Cronbach’s alpha 0.84 [33]
of Competence [32]	effectiveness	48	≥31 = high effectiveness	

Gray field indicates the scales/subscales forming the domain of negative emotions.

**Table 3:** Demographic characteristics in the identified two subgroups of the experimental group.

	Adverse PES	Favorable PES	
	Mean ± SD (Min–Max)	Mean ± SD (Min–Max)	*p* Adverse
	or Number (%)	or Number (%)	vs. Favorable
number	36	44	..
age (years)	30.83 ± 4.42 (23.00–39.00)	31.04 ± 4.75 (20.00–40.00)	0.83 ^!^
body height (m)	1.66 ± 0.05 (1.57–1.80)	1.68 ± 0.06 (1.55–1.80)	0.16 ^!^
body mass index (kg/m^2^)	27.35 ± 2.39 (23.03–32.83)	27.63 ± 2.57 (22.86–33.46)	0.61 ^!^
	secondary: 8 (22.22)	secondary: 6 (13.64)	
education level	high: 28 (77.78)	high: 38 (86.36)	0.32 ^†^
	yes: 6 (16.67)	yes: 8 (18.18)	
pregnancy planning	no: 30 (83.33)	no: 36 (81.82)	0.86 ^†^

^!^ Student’s *t*-test; ^†^ Chi^2^ test; PES—postnatal emotional status.

**Table 4:** Demographic characteristics of the experimental group and matched control group.

	Experimental	Control	
	Mean ± SD (Min–Max)	Mean ± SD (Min–Max)	*p* Experimental
	or Number (%)	or Number (%)	vs. Control
number	80 (100)	80 (100)	..
age (years)	30.95 ± 4.58 (20.00–40.00)	29.99 ± 4.24 (19.00–40.00)	0.17 ^!^
body height (m)	1.67 ± 0.06 (1.55–1.80)	1.67 ± 0.05 (1.52–1.78)	0.61 ^!^
body mass index (kg/m^2^)	27.51 ± 2.48 (22.86–33.46)	27.04 ± 3.91(20.41–37.10)	0.36 ^!^
postnatal emotional	adverse: 36 (45)	adverse: 36 (45)	
status changes	favorable: 44 (55)	favorable: 36 (55)	..
	vocational: 0 (0.00)	2 (2.50)	
	secondary: 14 (17.50)	secondary: 14 (17.50)	
education level	high: 66 (82.50)	high: 64 (80.00)	0.25 ^†^
	yes: 14 (17.50)	yes: 16 (17.50)	
pregnancy planning	no: 66 (82.50)	no: 64 (82.50)	0.68 ^†^

^!^ Student’s *t*-test; ^†^ Chi^2^ test.

**Table 5:** Results for the **total** experimental and control groups.

			*p*		*p*	*p* Experimental
		Experimental	1 vs. 2	Control	1 vs. 2	vs. Control
	1	8.75 ± 4.99 [0–28]		9.21 ± 4.02 [0–18]		0.15 ^†^
	2	7.11 ± 4.25 [0–18]	0.01 ^*	9.03 ± 4.64 [0–22]	0.73 ^!^	0.01 ^†^*
EPDS	Δ	−1.64 (−2.80–−0.47)		−0.19 (−1.25–0.88)		0.07 ^†^
	1	6.40 ± 4.06 [0–15]		5.20 ± 3.16 [0–12]		0.12 ^†^
	2	4.30 ± 3.04 [0–15]	<0.01 ^*	5.35 ± 3.23 [0–13]	0.76 ^	0.03 ^†^*
HADS (dep)	Δ	−2.10 (−3.01–−1.19)		0.15 (−0.52–0.82)		<0.01 ^†^*
	1	4.69 ± 3.15 [0–12]		3.76 ± 3.14 [0–12]		0.06 ^†^
	2	2.59 ± 2.48 [0–8]	<0.01 ^*	4.72 ± 3.27 [0–14]	0.02 ^*	<0.01 ^†^*
HADS (anx)	Δ	−2.10 (−2.83–−1.37)		0.96 (0.22–1.71)		<0.01 ^!^*
	1	23.44 ± 6.79 [13–49]		22.55 ± 7.30 [10–39]		0.47 ^†^
	2	19.35 ± 5.98 [7–38]	<0.01 ^*	22.07 ± 7.34 [8–38]	0.76 ^	0.02 ^†^*
PANAS (neg)	Δ	−4.09 (−5.85–−2.32)		−0.47 (−2.16–1.21)		<0.01 ^†^*
	1	37.45 ± 5.72 [22–49]		36.80 ± 6.42 [22–48]		0.50 ^!^
	2	41.50 ± 4.94 [30–50]	<0.01 ^*	37.58 ± 6.02 [21–49]	0.29 ^!^	<0.01 ^†^*
PANAS (pos)	Δ	4.05 (2.82–5.28)		0.78 (−0.66–2.21)		<0.01 ^!^*
	1	34.90 ± 5.58 [22–46]		35.26 ± 6.85 [16–48]		0.55^!^
	2	38.04 ± 6.26 [21–49]	<0.01 ^*	35.21 ± 5.66 [24–48]	0.99 ^	<0,01 ^†^*
PSOC (sat)	Δ	3.14 (1.42–4.85)		−0.05 (−1.38–1.28)		<0.01 ^!^*
	1	25.63 ± 4.74 [17–38]		27.71 ± 5.07 [16–41]		0.01 ^†^*
	2	29.53 ± 5.62 [19–43]	<0.01 ^*	27.86 ± 5.75 [18–40]	0.94 ^	0.03 ^†^*
PSOC (effect)	Δ	3.90 (2.81–4.99)		0.15 (−1.28–1.58)		<0.01 ^!^*

* Statistically significant; ^!^ Student’s *t*-test; ^†^ Mann–Whitney test; ^ Wilcoxon test. EPDS—Edinburgh Postnatal Depression Scale; HADS—Hospital Depression and Anxiety Scale (dep—depression subscale, anx—anxiety subscale); PANAS—Positive and Negative Affect Schedule (neg—negative affect subscale, pos—positive affect subscale); PSOC—Parenting Sense of Competence (sat—satisfaction subscale, effect—effectiveness subscale). 1/2—initial/final measurement (mean ± standard deviation [min–max] (in points)); Δ—difference score: final minus initial measurement (mean (95% confidence interval)). Gray field indicates the domain of negative emotions.

**Table 6:** Results for the averse trajectory of postnatal emotional status changes subgroups of the experimental and control groups.

			*p*		*p*	*p* Experimental
		Experimental	1 vs. 2	Control	1 vs. 2	vs. Control
	1	6.28 ± 2.80 [0–12]		9.42 ± 4.37 [0–18]		<0.01 ^!^*
	2	7.69 ± 4.41 [1–18]	0.04 ^!^*	12.28 ± 3.44 [6–22]	<0.01 ^*	<0.01 ^†^*
EPDS	Δ	1.42 (0.01–2.82)		2.86 (1.34–4.39)		0.14 ^†^
	1	4.11 ± 3.27 [0–13]		5.42 ± 3.50 [0–11]		0.10 ^†^
	2	4.89 ± 3.33 [1–15]	0.06 ^	7.03 ± 3.12 [2–13]	<0.01 ^*	0.01 ^†^*
HADS (dep)	Δ	0.78 (−0.26–1.82)		1.61 (0.68–2.54)		0.35 ^†^
	1	2.64 ± 2.36 [0–10]		3.69 ± 3.24 [0–11]		0.26 ^†^
	2	2.89 ± 2.63 [0–8]	0.38 ^	6.94 ± 2.80 [1–14]	<0.01 ^*	<0.01 ^†^*
HADS (anx)	Δ	0.25 (−0.73–1.23)		3.25 (2.23–4.27)		<0.01 ^†^*
	1	21.17 ± 4.61 [13–29]		22.75 ± 7.74 [10–36]		0.51 ^†^
	2	21.61 ± 6.46 [12–38]	0.71 ^!^	27.92 ± 5.87 [16–38]	<0.01 ^*	<0.01 ^!^*
PANAS (neg)	Δ	0.44 (−1.99–2.88)		5.17 (3.57–6.76)		<0.01 ^!^*
	1	40.17 ± 4.66 [30–49]		38.06 ± 7.17 [22–48]		0.14 ^!^
	2	42.06 ± 5.12 [30–50]	0.02 ^!^*	34.50 ± 4.99 [21–42]	<0.01 ^!^*	<0.01 ^!^*
PANAS (pos)	Δ	1.89 (0.27–3.50)		−3.56 (−5.19–−1.92)		<0.01 ^!^*
	1	37.72 ± 4.21 [27–45]		36.36 ± 5.74 [27–47]		0.39 ^†^
	2	36.14 ± 6.79 [21–45]	0.19 ^	32.58 ± 3.64 [24–40]	<0.01 ^*	0.01 ^†^*
PSOC (sat)	Δ	−1.58 (−3.56–0.39)		−3.78 (−5.20–−2.36)		0.07 ^†^
	1	26.39 ± 4.24 [18–36]		28.57 ± 5.10 [16–41]		0.05 ^!^
	2	28.28 ± 5.11 [20–38]	0.01 ^!^*	24.97 ± 2.87 [20–32]	<0.01 ^*	<0.01 ^!^*
PSOC (effect)	Δ	1.89 (0.41–3.37)		−3.58 (−5.24–−1.93)		<0.01 ^!^*

* Statistically significant; ^!^ Student’s *t*-test; ^†^ Mann–Whitney test; ^ Wilcoxon test. EPDS—Edinburgh Postnatal Depression Scale; HADS—Hospital Depression and Anxiety Scale (dep—depression subscale, anx—anxiety subscale); PANAS—Positive and Negative Affect Schedule (neg—negative affect subscale, pos—positive affect subscale); PSOC—Parenting Sense of Competence (sat—satisfaction subscale, effect—effectiveness subscale). 1/2—initial/final measurement (mean ± standard deviation [min–max] (in points)); Δ—difference score: final minus initial measurement (mean (95% confidence interval)). Gray field indicates the domain of negative emotions.

**Table 7:** Results for the favorable trajectory of postnatal emotional status changes subgroups of the experimental and control groups.

			*p*		*p*	*p* Experimental
		Experimental	1 vs. 2	Control	1 vs. 2	vs. Control
	1	10.77 ± 5.49 [3–28]		9.05 ± 3.75 [1–17]		0.23 ^†^
	2	6.64 ± 4.11 [0–15]	**<0.01** ^*	6.36 ± 3.72 [0–15]	**<0.01** ^*	0.88 ^†^
EPDS	Δ	−4.14 (−5.56–−2.71)		−2.68 (−3.71–−1.65)		0.10 ^!^
	1	8.27 ± 3.69 [2–15]		5.02 ± 2.89 [0–12]		<0.01 ^!^*
	2	3.82 ± 2.73 [0–10]	**<0.01** ^*	3.98 ± 2.63 [0–11]	**0.01**^!^*	0.74 ^†^
HADS (dep)	Δ	−4.45 (−5.44–−3.47)		−1.05 (−1.87–−0.22)		<0.01 ^†^*
	1	6.36 ± 2.71 [1–12]		3.82 ± 3.09 [0–12]		<0.01 ^†^*
	2	2.34 ± 2.36 [0–8]	**<0.01** ^*	2.91 ± 2.41 [0–9]	**0.01** ^*	0.19 ^†^
HADS (anx)	Δ	−4.02 (−4.67–−3.37)		−0.91 (−1.61- −0.21)		<0.01 ^†^*
	1	25.30 ± 7.71 [15–49]		22.39 ± 7.01 [10–39]		0.10 ^†^
	2	17.50 ± 4.90 [7–27]	**<0.01** ^*	17.30 ± 4.3 [58–26]	**<0.01**^!^*	0.84 ^!^
PANAS (neg)	Δ	−7.80 (−9.76–−5.83)		−5.09 (−6.99–−3.19)		<0.01 ^†^*
	1	35.23 ± 5.59 [22–47]		35.77 ± 5.61 [25–48]		0.65 ^!^
	2	41.05 ± 4.80 [31–49]	**<0.01**^!^*	40.09 ± 5.65 [27–49]	**<0.01** ^*	0.55 ^†^
PANAS (pos)	Δ	5.82 (4.14–7.50)		4.32 (2.67–5.96)		0.20 ^!^
	1	32.59 ± 5.53 [22–46]		34.36 ± 7.59 [16–48]		0.21 ^!^
	2	39.59 ± 5.38 [24–49]	**<0.01** ^*	37.36 ± 6.13 [25–48]	**<0.01**^!^*	0.07 ^!^
PSOC (sat)	Δ	7.00 (4.90–9.10)		3.00 (1.32–4.68)		<0.01 ^!^*
	1	25.00 ± 5.08 [17–38]		27.02 ± 5.01 [17–38]		0.04 ^†^*
	2	30.55 ± 5.86 [19–43]	**<0.01** ^*	30.23 ± 6.42 [18–40]	**<0.01** ^*	0.03 ^†^*
PSOC (effect)	Δ	5.55 (4.10–6.99)		3.20 (1.39–5.02)		0.04 ^!^*

* Statistically significant; ^!^ Student’s *t*-test; ^†^ Mann–Whitney test; ^ Wilcoxon test. EPDS—Edinburgh Postnatal Depression Scale; HADS—Hospital Depression and Anxiety Scale (dep—depression subscale, anx—anxiety subscale); PANAS—Positive and Negative Affect Schedule (neg—negative affect subscale, pos—positive affect subscale); PSOC—Parenting Sense of Competence (sat—satisfaction subscale, effect—effectiveness subscale) 1/2—initial/final measurement (mean ± standard deviation [min–max] (in points)); Δ—difference score: final minus initial measurement (mean (95% confidence interval)) Gray field indicates the domain of negative emotions.

## Data Availability

The data presented in this study are available on request from the corresponding author due to legal reasons.

## Abbreviations

The following abbreviations are used in this manuscript:

PES	Postnatal emotional status
PPD	Postpartum depression
EPDS	Edinburgh Postnatal Depression Scale
HADS	Hospital Depression and Anxiety Scale
PANAS	Positive and Negative Affect Schedule
PSOC	Parenting Sense of Competence
